# Vaccines After an Emergency Use Authorization (EUA): Modern Evidence Generation Approaches

**DOI:** 10.1007/s43441-021-00290-z

**Published:** 2021-04-22

**Authors:** Névine Zariffa, Estelle Russek-Cohen

**Affiliations:** 1 NMD Group LLC, Philadelphia, PA USA; 2 ERCStatLLC, Rockville, MD USA

## Abstract

Every medical product requires additional study even after regulatory approval. We highlight several lines of enquiry to advance our understanding of COVID19 vaccines post authorization: identifying key population segments warranting more study, assessment of efficacy, and of safety data, harmonization of data relating to immune response and developing mechanisms for data and knowledge sharing across countries. We show how innovative trial designs and sources from real world data play a critical role in generating evidence.

## Introduction

Effective vaccination strategies play a key part in halting the impact of the COVID19 pandemic. Thanks to the commitment and resources of the biotechnology, pharmaceutical, academic, government and philanthropic sectors, several vaccines are being studied in large randomized controlled trials in record time. The primary objectives of these trials are to study the effect of the vaccine in reducing the number of overt COVID19 cases relative to placebo [1]. Results from interim analyses have been presented in publications [2–4] and briefing documents for FDA Advisory Committees [5, 6]. As of December 2020, vaccinations have begun in several countries following authorization by Health Authorities.

Lipsicht and Dean [7] highlight many open questions that remain after these initial trials. We identify three key scientific questions: population segments, durability of protection, and safety monitoring. We briefly describe the context for each question, noting what can be reliably inferred from the trials and what remains to be addressed in the post-marketing (PM) setting. We then assess experimental approaches, existing data sources, analysis and interpretation modalities.

## Population Segments

We define population segments as demographic subgroups (eg age, gender, race), comorbidities (eg prior cancer, diabetes, obesity), community factors (eg community living, schools), and COVID19 risk factors (eg healthcare workers, first responders). While the vaccine trials are large, the primary comparisons for efficacy rely on the number of COVID19 cases as defined in the protocol, typically about 200 events. Interrogation within subsets, with even fewer events, creates more uncertainty in the statistical assessments. In addition to evaluating short-term efficacy and safety across subgroups, durability of effect and other research questions need to be addressed in population segments. While manufacturers made a concerted effort to enroll a diverse population, especially minority groups, some groups were underrepresented as compared to the COVID19 incidence rate in these segments. The trials were not designed to assess the role comorbidities and risk factors may play in efficacy and safety. We are left to provide this information in the PM phase.

Thus, our first recommendation is for a set of population segments to be developed and defined to facilitate future research across data sources. There will be a need to prioritize which segments warrant additional study. A second recommendation is to harmonize the definition and ascertainment of key efficacy and safety endpoints. Efficacy assessments should be able to link back to the trial endpoint definitions and expand upon these as relevant. For safety, common definitions and ascertainment strategies that facilitate integration of knowledge generated across all data sources would be valuable.

## Durability of Protection

Durability of protection can be assessed using incident disease of COVID19 as used in the current vaccine trials or using a marker which is associated with protection. Combining data from multiple sources to establish a robust Correlate of Protection (CoP) is the preferred approach [8].

Having a CoP will greatly advance vaccine development. As the incidence of COVID19 drops because of effective vaccines, the next generation of vaccines could be developed based on the CoP in relatively small trials. A CoP can be used to establish duration of immunity and decide if and when a booster is needed.

There are still open questions on the nature of protection beyond just durability. In spite of having several successful highly effective vaccines authorized to date, none of the pivotal trials achieved 100% vaccine effectiveness, none evaluated sterilizing immunity (namely can a successfully vaccinated individual still infect others). Each vaccine would need to be separately evaluated if these limitations are of interest but none are required to achieve full approval from a regulatory authority.

Our third recommendation is for a full set of endpoints related to immunity to be defined (with appropriate descriptive assay information) and available for health systems and future trials to deploy in a structured way so key questions can be addressed.

## Safety

The vaccine trials have enrolled 30,000 to 44,000 participants with interim results when median exposure of 2 months had been achieved. Given the usual safety data collection in vaccine trials, characterization of the vaccines’ short-term safety data for common effects (eg rate greater than 0.1%) is reasonable. What remains are rare events (such Guillaume Barre Syndrome which occurs at a rate of 0.0001%) and long-term safety events not immediately recognized as vaccine related (eg pregnancy outcomes). Since new modalities of vaccine constructs are at play, there may be unanticipated safety signals that will require clear and quick adjudication.

There are established mechanisms for PM surveillance in the US and other countries but given the vaccine is to be distributed in an unusual way (eg via pharmacies, places of work, community settings etc.), traditional PM approaches [9] may require rethinking and enhancements. Pharmaceutical sponsors and Regulatory authorities around the world are expanding their approaches which will yield important safety information.

Our fourth recommendation is for a clear communication mechanism to be established both at the country- and global-level for initial signals and for validated findings. All four Primary Recommendations are listed in Table 1.

**Table 1:** Primary Recommendations

1	Identify population segments of primary interest to be used in research across data sources; this facilitates gathering and synthesis of information across data sources
2	Harmonize definitions and ascertainment of key efficacy and safety endpoints to enable aggregation of data and comparisons from one health data source to next as well as with clinical trials
3	Develop a full set of endpoints related to immune response, including meta-data describing assay characteristics, for both health systems and future clinical trials to deploy in a structured way
4	Establish a clear mechanism within countries and at the global level to review and communicate knowledge as it evolves

## Modern approaches for evidence generation

These factors suggest a cross-vaccine, cross-regulatory initiative is required for enhancing public confidence and addressing efficacy, durability and safety of vaccines in the worldwide population, and in specific segments. Given the differential between the size of the vaccine trials and the much larger global population, there is no doubt that many residual uncertainties can only be addressed in the PM setting. That said, both traditional and innovative randomized trials as well as real world evidence (RWE) approaches will be required.

## Randomized Clinical Trials

Traditional trials or registries and immunobridging studies should be considered prior to full vaccine deployment and initiated quickly for pediatric populations, pregnant women, minority groups, and so on.

In addition to these targeted studies, one could envisage platform trials [10, 11] to compare the efficacy of various vaccines in segments of the population. These may be adaptive so that if several vaccines can be shown to be ‘equivalent’ in a population segment, they can be dropped from that part of the study. Likewise, if one is found to be sub-standard, that arm can be terminated, and the results communicated quickly. These designs would be novel since most platform trials focus on determining superiority of treatments rather than equivalence.

Cluster randomized trials (CRT) could be considered for the direct comparison of vaccines. In such trials, regions or institutions are the unit of randomization though individual subject informed consent is still required [12]. These are easier to implement than traditional trials and given the distribution complexities of individual manufacturer’s vaccines, could offer a substantial advantage. The fundamental unit of randomization may be a nursing home or even a city, but studies must have a reasonable number of clusters. The usual assumption is that within-cluster correlation is small but still needs to be considered in the analysis. CRTs can also be employed to evaluate educational or dose schedule strategies.

Last, one could envisage trials that include randomization to one vaccine or another as described here with follow up from real world data (RWD) sources to supplement the traditional trial data collection. This could include patient reported outcomes for symptom assessments.

## Real World Data

In general, RWD sources encompass registries, claims data, health care records (hospital or clinical settings) and data sets that link the underlying data for a patient in multiple environments.

We begin with safety. Well-established channels exist for the evaluation of comparative effectiveness and for monitoring of safety in the PM setting using RWD [13, 14]. These can be leveraged in the case of COVID19 vaccines—and must be augmented given the need for transparent and clear information to allow the public to form its view on the level of confidence it will attach to the vaccines.

## RWD for Safety Monitoring

Safety monitoring in the US relies on both manufacturer’s specific PM surveillance proposals and established regulatory data systems such as SENTINEL and the vaccine adverse event reporting system (VAERS). Pfizer and Moderna presented their respective PM approaches at FDA Advisory Committees [5, 6]. FDA has since provided PM surveillance protocols for review via BEST [15, 16], though this must be augmented with process and communication considerations. Alignment with the WHO PV guidance would be helpful for a global approach to safety [17]. Other considerations for the worldwide setting are discussed by Chandler [18]. The example of a unified detection and adjudication process deployed for H1N1 influenza vaccine in 2009 could be used as a foundation, updated with current technologies and tools for harnessing RWD at scale.

Since COVID19 vaccines will be administered to a very large population quickly, the opportunity to enhance our understanding of even very rare events (say 1 in 100,000) is more substantial than for any prior vaccination effort. There are foundational elements required immediately (see Table 2).

**Table 2:** Considerations for Safety Evaluation in Real World Data Sources

Overall Considerations	Implementation Considerations
1. *Vaccine Exposure* Ability to identify timing and manufacturer for each vaccine administration. This is a foundational and critical element for any work in observational data sources	1. In countries with National Health Systems, this is achievable. In the US, the initial AMA codes [19] must be adhered to and captured in such a way as to ‘linked’ to other data from those vaccinated
2. *Data Linkage* Ability to link to EMR (non-serious AEs) and EHR (serious AEs) for a large representative portion of the population	2. The Veteran’s Administration and the VSD [20] system fulfill this requirement though the VA tends to focus on older, mostly male subjects. Other data sets are required to cover the rest of the affected population (eg Health Verity)
3. *Patient Engagement* Use of “direct from patients” technologies for symptoms and daily evolution	3. Examples of registries that could be adapted are CARE (from IQVIA) and HERO-TOGETHER (Duke). Emphasis on engagement, adherence, and completeness of data for the general public should be incorporated into the platforms
4. *Learn and Confirm mindset* Updating passive safety surveillance systems to enhance automated reporting of COVID19 vaccine adverse events [21]	4. Following an initial period where COVID19 safety signals are identified and prioritized, algorithms will be required to allow automated and accurate assessment of any safety events that are not routinely available in the surveillance system

Statistical Considerations	Implementation Considerations
5. *Comparator cohorts* Using pre-COVID19 populations to establish background rates of events of interest in the general population is important. From such data sets, risk matched cohorts for specific comparisons can be created	5. Having each manufacturer establish their own approach may be helpful initially to establish best practice. The stated intent would be to achieve a unified approach used by all manufacturers in short order
6. *Impact of data considerations on analysis methods* Data sources will vary as to the completeness and specificity of their data	6. Matching the specific safety question under study to appropriate data sets is necessary. Passive surveillance sources may require distinct statistical analysis strategies
7. *Using Unstructured Data* When events of interest are not readily available, unstructured data fields may be required in aggregate with other data fields	7. Natural language processing can be used to combine information from text fields with that in structured data fields to provide an approximation for these events [22]
8. *Nature of statistical inference* For AEs that occurs in near proximity to administration of the COVID19 vaccine, self-control methods may be used. For longer-term AEs, the choice of comparator, establishing the risk set will be required [23]	8.a Multiple statistical approaches are used, each with strengths and limitations 8.b There is no one approach that is a gold standard

Process Considerations	Implementation Considerations
9. Frequent *meetings of experts* in each region of the globe reviewing data, adjudicating signals and reporting their findings to other such expert groups globally	9.a Regulators have compared safety concerns for influenza vaccines so a precedent exists 9.b Regular communications and postings after each meeting for the public will be critical given the ambition to vaccinate most of the world’s population
10. A *central data center*, preferably already in place, could be used to ensure all countries have access to relevant safety data for their own interrogation	10. The US, the EMA and the WHO use the Uppsala center in Sweden to collect passive surveillance safety data (VIGIBASE system) including data from the US VAERs system [24]

## RWD sources for evaluation of durability and efficacy in segments of the population

Similar considerations as presented for safety apply for efficacy and durability assessments. Studying these in parallel in the same sources if appropriate allows direct benefit-risk characterization.

Some segments of interest, (e.g., healthcare workers) are especially motivated to volunteer for registries or targeted linked systems [25]. Pregnant women are typically studied in national registries or as a PM requirement. Registries for subjects that are on a specific medication or have a chronic illness that puts them at increased risk for a reaction to a vaccine could provide quick information on such reactions. In all cases, planning what information is gathered and when offers scientists and regulators a tremendous asset. While registries are not randomized, they allow for the study of risk factors and other population covariates, they can establish temporal effects and provide other insights based on cohesive and rich data collection strategies.

Some of the most important open questions including whether one vaccine is preferred over another in a particular segment, e.g., the elderly, could be studied in randomized trials, but it’s more likely that initial observations will come from RWD. Since the likely questions are already established, one could envisage multiple parallel interrogations such as deployed in the OHDSI network [26, 27], and more recently in the Reagan-Udall/Friends of Cancer Research [28] activities. Since gold standard approaches are not yet established for RWE, having a plurality of approaches initially would be informative. In addition, having set venues where RWE researchers debate their protocols prior to analysis, and review results in concert with full transparency will set a good standard for reliability of results [29].

## Conclusion

The development of COVID19 vaccines has been an exceptional achievement. The initial randomized controlled trials were designed and executed to yield credible and clear results which enabled emergency use authorization around the world. The important questions that come next will be answered using other data sources.
